# Prevention and Intervention Studies with Telmisartan, Ramipril and Their Combination in Different Rat Stroke Models

**DOI:** 10.1371/journal.pone.0023646

**Published:** 2011-08-25

**Authors:** Christa Thoene-Reineke, Kay Rumschüssel, Kristin Schmerbach, Maxim Krikov, Christina Wengenmayer, Michael Godes, Susanne Mueller, Arno Villringer, Ulrike Steckelings, Pawel Namsolleck, Thomas Unger

**Affiliations:** 1 Center for Cardiovascular Research (CCR)/Institute of Pharmacology, Charité – Universitätsmedizin Berlin, Berlin, Germany; 2 Forschungseinrichtung für Experimentelle Medizin, Charité – Universitätsmedizin Berlin, Berlin, Germany; 3 Center for Stroke Research Berlin, Charité – Universitätsmedizin Berlin, Berlin, Germany; 4 MPI für Kognitions- und Neurowissenschaften, Leipzig, Germany; Julius-Maximilians-Universität Würzburg, Germany

## Abstract

**Objectives:**

The effects of AT1 receptor blocker, telmisartan, and the ACE inhibitor, ramipril, were tested head-to head and in combination on stroke prevention in hypertensive rats and on potential neuroprotection in acute cerebral ischemia in normotensive rats.

**Methods:**

Prevention study: Stroke-prone spontaneously hypertensive rats (SHR-SP) were subjected to high salt and randomly assigned to 4 groups: (1) untreated (NaCl, n = 24), (2) telmisartan (T; n = 27), (3) ramipril (R; n = 27) and (4) telmisartan +ramipril (T+R; n = 26). Drug doses were selected to keep blood pressure (BP) at 150 mmHg in all groups. Neurological signs and stroke incidence at 50% mortality of untreated SHR-SP were investigated.

Intervention study: Normotensive Wistar rats were treated s.c. 5 days prior to middle cerebral artery occlusion (MCAO) for 90 min with reperfusion. Groups (n = 10 each): (1) sham, (2) vehicle (V; 0,9% NaCl), (3) T (0,5 mg/kg once daily), (4) R (0,01 mg/kg twice daily), (5) R (0,1 mg/kg twice daily) or (6) T (0,5 mg/kg once daily) plus R (0,01 mg/kg twice daily). Twenty-four and 48 h after MCAO, neurological outcome (NO) was determined. Forty-eight h after MCAO, infarct volume by MRI, neuronal survival, inflammation factors and neurotrophin receptor (TrkB) were analysed.

**Results:**

Stroke incidence was reduced, survival was prolonged and neurological outcome was improved in all treated SHR-SP with no differences between treated groups. In the acute intervention study, T and T+R, but not R alone, improved NO, reduced infarct volume, inflammation (TNFα), and induced TrkB receptor and neuronal survival in comparison to V.

**Conclusions:**

T, R or T+R had similar beneficial effects on stroke incidence and NO in hypertensive rats, confirming BP reduction as determinant factor in stroke prevention. In contrast, T and T+R provided superior neuroprotection in comparison to R alone in normotensive rats with induced cerebral ischemia.

## Introduction

Several recent clinical trials have demonstrated the efficacy of angiotensin AT1 receptor blockers (ARBs) and angiotensin-converting enzyme inhibitors (ACEI) in primary and secondary prevention of stroke in patients with hypertension and/or high cardiovascular risk [1]–[6]. Whereas the antihypertensive actions of these drugs are thought to contribute to their stroke-preventing features, the question as to potential blood pressure-independent neuroprotective effects of renin-angiotensin-system (RAS) inhibitors under cerebral ischemia still remains open, especially since clinical drug trials on stroke, for obvious reasons, usually address stroke incidence alone and rarely issues of neuroprotection.

ACE inhibitors and angiotensin receptor antagonists both target the RAS, though in different ways. ACE inhibitors never block the RAS completely. Even when ACE is inhibited by more than 90%, there will still be some angiotensin II production by other enzymes that compensate for the lack of ACE activity. Angiotensin receptor antagonists, on the other hand, feature a more specific mechanism since they selectively block the angiotensin receptor subtype-1 (AT1), which is responsible for most of the deleterious effects of angiotensin II, and expose the unblocked angiotensin receptor subtype-2 (AT2) to increased angiotensin II concentrations. Published data suggest that the AT2 receptor can counterbalance the effect of the AT1 receptor *in vitro* as well as *in vivo* [7]. It has also been speculated that combining ACE inhibition and AT1 receptor blockade could have advantages over each therapeutic principle alone [8] but with respect to neuroprotection in cerebral ischemia, this hypothesis has not yet been tested.

In the present study, we addressed two questions: First, in a well established hypertensive animal model with cerebrovascular disease and a high incidence of stroke, the salt-fed stroke-prone spontaneously hypertensive rat (SHR-SP), we asked whether a similar inhibition of the RAS as evidenced by equal blood pressure reductions by ACE-inhibition, AT1 receptor blockade, or their combination would engender comparable effects on stroke incidence and stroke-related morbidity and mortality (prevention study).

Second, in normotensive rats, we evaluated the potential blood pressure-independent neuroprotective effects and mechanisms of subantihypertensive treatment regimens involving comparable blockade of ACE or AT1 receptors or combined treatment with both of these principles (acute intervention study).

We used telmisartan as ARB and ramipril as ACEI since these drugs have recently been used alone and in combination in a large clinical trial involving stroke prevention in patients with high cardiovascular risk [6]. In the rat, both drugs have been demonstrated to cross the blood-brain barrier with telmisartan having a higher propensity to enter the brain than ramipril [9], [10].

## Materials and Methods

### Animals and treatment

Rats were kept in a SPF (specific pathogen free) barrier under standardized conditions with respect to temperature and humidity, and were housed on a 12 h light/12 h dark cycle in groups of 4–5 with food and water *ad libitum*. Animal housing, care, and applications of experimental procedures were approved by State Office of Health and Social Affairs Berlin, Germany (project IDs G0034/06 and G0088/04).

The human end point was set by a neurological score of 3.

### Prevention study

**Figure 1:**
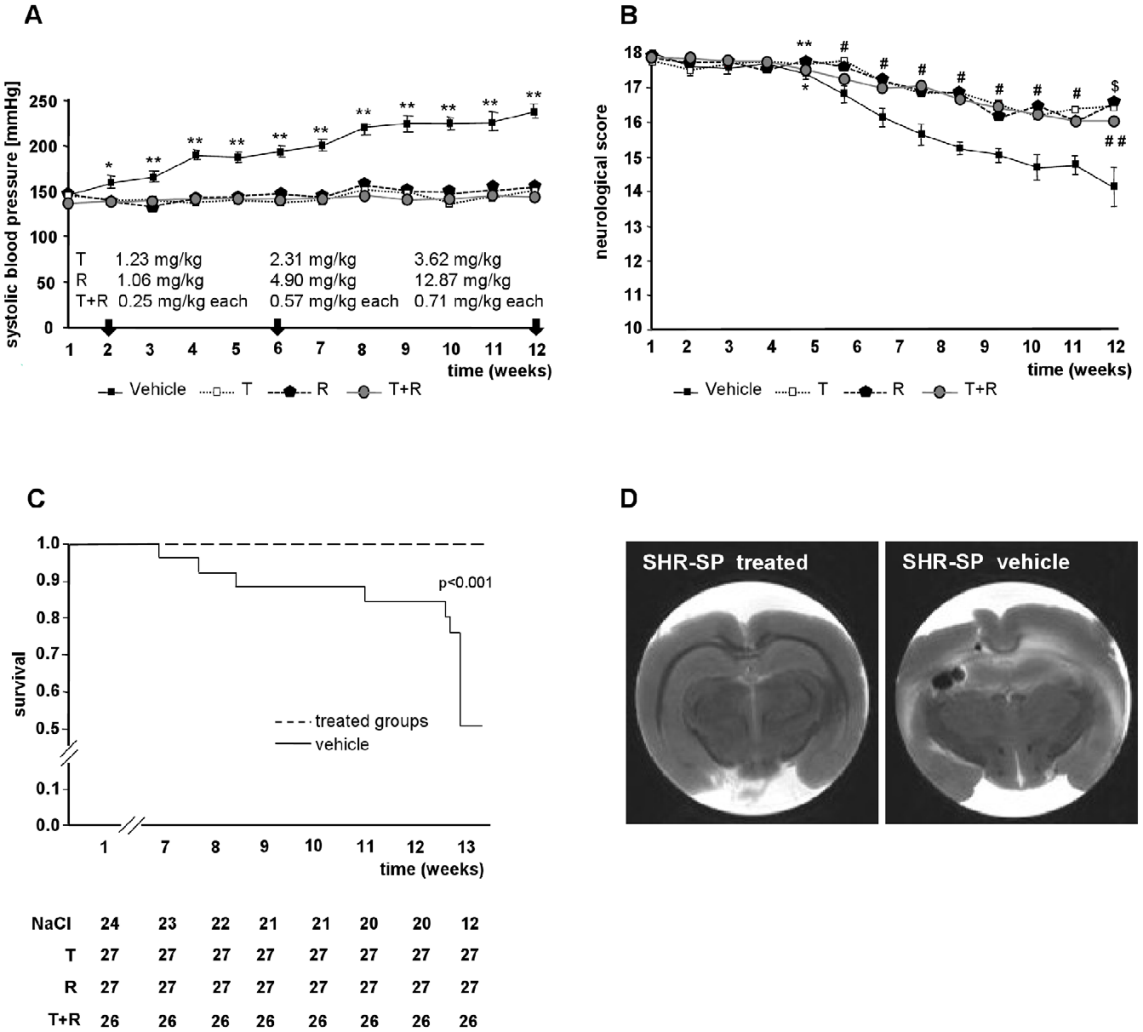
Prevention study SHR-SP. A) Development of systolic blood pressure in hypertensive treated and untreated rats measured by tail cuff method. Results area expressed as mean SEM. *p<0.05 or **p<0.01 significant differences between treatment groups and vehicle. B) Effect of vehicle, telmisartan, ramipril or telmisartan plus ramipril supplemented in drinking water on neurological deficits in salt-loaded, stroke-prone spontaneously hypertensive rats. *p<0.05 telmisartan treated group; **P<0.01 ramipril treated group vs vehicle group, 13 weeks of age. *^#^p*<0.01 telmisartan, ramipril and telmisartan + ramipril treated groups vs vehicle group, 14–20 weeks of age. *^##^p*<0.05 telmisartan + ramipril treated group; *^$^p*<0.01 telmisartan, ramipril treated groups vs vehicle group, 21 weeks of age. C) Survival rate in salt-loaded, stroke prone spontaneously hypertensive rats (SHR-SP). Nine week-old SHR-SP were treated with vehicle, telmisartan, ramipril or telmisartan plus ramipril supplemented in drinking water. Figures under the curve show number of surviving animals. Mortality was compared among groups using Kaplan Meier analysis of survival followed by log-rank test. *p<0.001; telmisartan, ramipril and telmisartan + ramipril treated groups vs vehicle group. D) Representative picture of SHR-SP with vehicle and stroke and with treatment without stroke.

Male SHR-SP (n = 104), aged 7 weeks, were obtained from Charles River Laboratories (Sulzfeld, Germany). Baseline measurements (MRI, blood pressure by tail cuff method) and neurological score were performed at the age of 9 weeks. Subsequently, all rats were switched to a high salt diet (RMH-TM rat chow; protein 22.5%; fat 4.8%; potassium 0.85%; sodium 0.40%; Hope Farms, Sniff, Soest, Germany) and 1% NaCl (170 mmol/L) in the drinking water in order to accelerate the appearance of cerebrovascular events [11]. With the beginning of the high salt diet, SHR-SP were randomly assigned to four different treatment groups: group 1 served as control (untreated; *n* = 24), group 2 treated with the angiotensin AT1 receptor blocker telmisartan in the drinking water (initial dose T; 1.23 mg/kg bwt; *n* = 27), group 3 treated with the ACE-inhibitor ramipril in drinking water (initial dose R; 1.06 mg/kg bwt; *n* = 27), and group 4 was treated with a combination of telmisartan and ramipril in the drinking water (initial dose T; 0.25 mg/kg bwt plus R, 0.25 mg/kg bwt; n = 26) (Figure S1A). Individual drug doses were selected on the basis of achieving equal blood pressure (150 mmHg) throughout the protocol (Figure 1A).

Systolic blood pressure was measured weekly in trained, conscious and pre-warmed rats by tail cuff method using a model from ADInstruments with PowerLab software [12]. Rats were screened daily for neurological disorders by the scoring system of Garcia et al. [13]. The protocol was terminated at 50% mortality in the untreated SHR-SP group (Figure 1A–D).

### Acute intervention study

**Figure 2:**
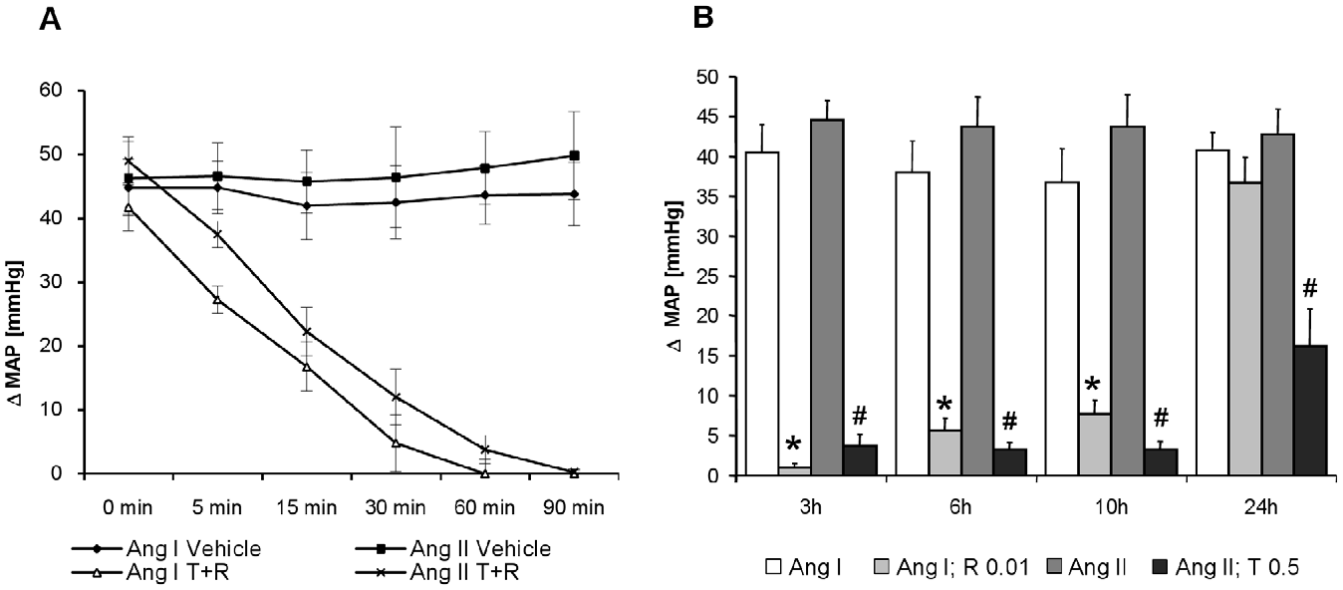
Intervention study. A) Effects of combination of ramipril (0.01 mg/kg bw) and telmisartan (0.5 mg/kg bw) or vehicle administered subcutaneously on the pressor responses to i.v. injected angiotensin I (150 ng/kg bw) or angiotensin II (50 ng/kg bw; n = 7). B) Duration of the pressor responses of selected doses of ramipril (0.01 mg/kg bwt) and telmisartan (0.5 mg/kg bwt) administered subcutaneously induced by angiotensin I (150 ng/kg bwt) and angiotensin II (50 ng/kg bwt) injected intravenously (n = 7 per group); *: *p*<0.05 vs. ANG II; #: *p*<0.05 vs. ANG I.

In a pilot study, male normotensive Wistar rats (160–200 g, HARLAN Winkelmann, Borchen, Germany) were randomly divided in several groups (Ang I+0,9% NaCl; Ang I+R 0.01 mg/kg; Ang I+T 0.05+R 0.01; Ang II+0,9% NaCl; Ang II+T 0.05 mg/kg, Ang II T 0.05+R 0.01; n: 5 per group) to determine the appropriate treatment doses of the comparators (Figure 2A–B; Figure S2A–B).

Based on the above dose finding, further animals were randomly allocated to five different treatment groups (n = 10 each): (1) sham-operated, vehicle (V; 0.9% NaCl once daily) treated, (2) stroke V (0.9% NaCl once daily) treated, (3) stroke T (0.5 mg/kg once daily) treated, (4) stroke R (0.01 mg/kg twice daily) treated, (5) stroke R (0.1 mg/kg twice daily) treated or (6) stroke T (0.5 mg/kg once daily) plus R (0.01 mg/kg twice daily). Subcutaneous (s.c.) drug treatment was begun 5-days prior to MCAO [14] (Figure S1B).

### Femoral artery and vein catheter in acute intervention study

Two days before blood pressure recordings (pilot study) and three days after initiation of treatment (main study), polyethylene catheters (PP-50) were inserted through the femoral artery into the abdominal aorta and in the femoral vein. The arterial catheter was used for blood pressure measurement via a transducer using the software PowerLab by ADInstruments (Spechtbach, Germany). The femoral vein catheter was used for intravenous (i.v.) injections of angiotensin II (Ang II; 50 ng/kg bwt) and angiotensin I (Ang I; 150 ng/kg bwt), respectively [15].

### Determination of equipotent doses of telmisartan, ramipril and their combination in vivo for acute intervention study

On day two after catheterization, five animals per group were placed into individual cages and connected via the arterial catheter to a transducer for hemodynamic measurements and, via the venous line, to a microsyringe for small volume injections. After baseline blood pressure recordings for 30 min, animals received an intravenous (i.v.) bolus injection of Ang II or Ang I, respectively. Rats were then treated s.c. with NaCl solution, telmisartan or ramipril or their combination at different doses. The pressor responses to i.v. bolus injections of Ang II (telmisartan group or combination group) or Ang I (ramipril group or combination group) were recorded at time intervals of 5, 15, 30, 60 and 90 min and 3, 6 and 10 hours after s.c. vehicle, telmisartan or ramipril or their combination.

Bolus i.v. injections of Ang II or Ang I, five, 15, 30, 60 and 90 min after s.c. treatment with vehicle elicited consistent increases in mean arterial pressure of about 40 mmHg. Subcutaneous injection of telmisartan prior to Ang II dose- and time-dependently attenuated the pressor responses to the peptide. At 0.1 mg/kg bwt, telmisartan was not effective, whereas at 0.5 mg/kg bwt, telmisartan completely inhibited the pressor responses to Ang II within 30 minutes of s.c. administration. Based on these findings, a telmisartan dose of 0.5 mg/kg bwt was determined to be used in further experiments (Figure S2A).

The corresponding dose of ramipril, established by the same criteria as above (with exception that Ang I instead of Ang II was used as the i.v. pressor agent) was 0.01 mg/kg bwt. This dose blocked the pressor responses to i.v. Ang I by 90–100% from 30 to 90 min (Figure S2B).

The inhibition of the respective Ang II- and Ang I pressor responses with the above doses of telmisartan and ramipril after 3, 6 and 10 hours are shown in Figure 2A. Both compounds were inhibitory for 10 hours. Thereafter, ramipril lost effectiveness faster than telmisartan (Figure 2B). Based on these findings, a treatment protocol with twice daily s.c. injections was established for ramipril to guarantee continuous 24–hour RAS blockade *in vivo*.

Since inhibition of the brain RAS inside the blood-brain barrier (BBB) has been demonstrated to be neuroprotective under ischemic conditions [14]–[18] and since peripherally administered ramipril, at the dose established above, may penetrate the BBB less readily than telmisartan [9], [10], we introduced an additional group of rats pre-treated s.c. with ramipril at 0.2 mg/kg bwt (ramipril high dose) to allow for better comparison between drugs.

### Middle cerebral artery occlusion with reperfusion

**Figure 3:**
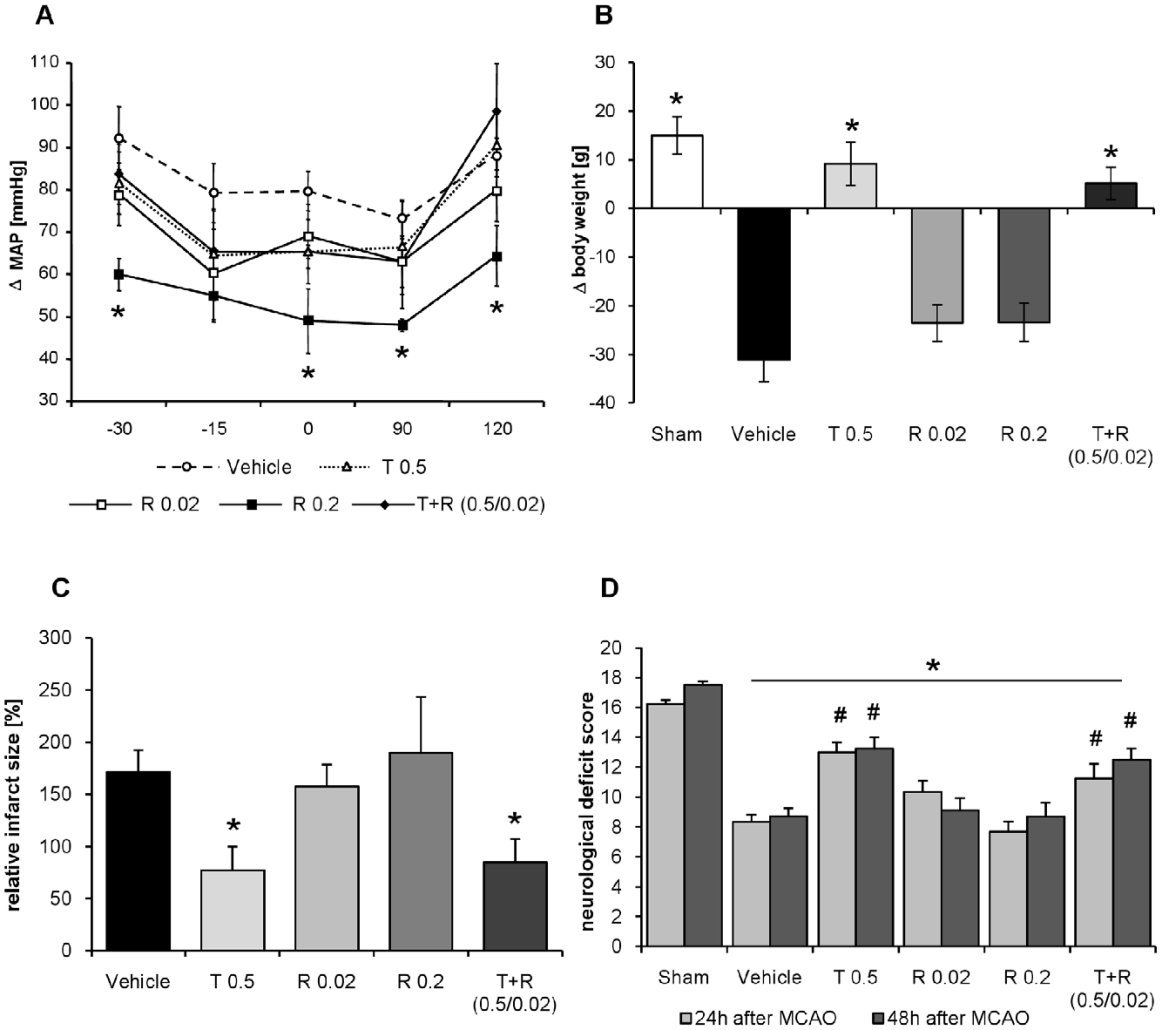
Intervention study. A) Effect of 5 day pretreatment with telmisartan (0.5 mg/kg bwt), ramipril (0.02 mg/kg bwt and 0.2 mg/kg bwt) and their combination (T 0.5 mg/kg bwt, R 0.02 mg/kg bwt) administered subcutaneously on mean arterial blood pressure (mmHg) before, during and after MCAO. Baseline (−30), initiation of anesthesia (−15), start of MCAO (0), 90 min. of occlusion (+90) and 30 min. of reperfusion (+120) (n = 7 per group); *: *p*<0.05 vs. Vehicle. B) Effects of 5 day pretreatment with ramipril, telmisartan and their combination administered subcutaneously at established doses on body weight gain 48 h after MCAO. Columns from the left: sham, vehicle, telmisartan 0.5 mg/kg (T 0.5), ramipril 0.02 mg/kg (R 0.02), ramipril 0.2 mg/kg (R 0.2) and combination of telmisartan 0.5 mg/kg and ramipril 0.02 mg/kg (T+R 0.5/0.02), n = 7 per group. *p<0.05 vs. vehicle C) Effect of 5 day pretreatment with ramipril, telmisartan and their combination on infarct volume. Columns from the left: vehicle, telmisartan 0.5 mg/kg (T 0.5), ramipril 0.02 mg/kg (R 0.02), ramipril 0.2 mg/kg (R 0.2) and combination of telmisartan 0.5 mg/kg and ramipril 0.02 mg/kg (T+R 0.5/0.02), n = 7 per group. *p<0.05 vs. stroke/vehicle D) Effect of 5 day pretreatment with ramipril, telmisartan and their combination on neurological deficit score 24 h (bright bars) or 48 h (dark bars) after MCAO. Columns from the left: sham, vehicle, telmisartan 0.5 mg/kg (T 0.5), ramipril 0.02 mg/kg (R 0.02), ramipril 0.2 mg/kg (R 0.2) and combination of telmisartan 0.5 mg/kg and ramipril 0.02 mg/kg (T+R 0.5/0.02), n = 7 per group. *p<0.05 vs. sham; #: *p*<0.05 vs. vehicle.

Five days after the beginning of treatment, focal cerebral ischemia was induced by right middle cerebral artery occlusion (MCAO) with subsequent reperfusion as described previously [16], [19]. Under general anaesthesia with chloralhydrate 400 mg/kg, the right cervical carotid bifurcation was exposed through a midline neck incision. A 4–0 silicon-coated nylon monofilament (Prolene, Ethicon GmbH, Norderstedt, Germany) was gently inserted through the proximal external carotid artery (ECA) into the internal carotid artery up to middle cerebral artery offshoot. After 90 minutes, the filament was withdrawn into the stump of the ECA to allow reperfusion (Figure S3A). Sham-operated rats underwent the same surgical procedures except that the occluding monofilament was not inserted. Cerebral blood flow (CBF) was monitored during surgical intervention with a probe attached to the skull above the supply territory of the MCA (2 mm caudal to bregma, 6 mm lateral to midline) by Laser-Doppler flowmetry (Periflux system 5000, PERIMED, Stockholm, Sweden). The procedure was considered successful, when an over 75% drop in CBF was observed after MCAO (Figure S3B). BP was measured before, during and after MCAO with atrial catheter (Figure 3A). Body weight was investigated before and after surgery (Figure 3B).

### T2-weighted Magnetic Resonance Imaging

Magnetic resonance imaging (MRI) was performed 48 hours after MCAO on a 7 T Bruker scanner (Pharmascan 70/16 AS, Bruker Biospin, Ettlingen, Germany) in isoflurane anaesthesia. Cerebral ischemic areas were visualized with a T2-weighted; fat suppressed 2D turbo spin-echo sequence (TR 5218.7 ms; TE_eff_ 65.2 ms, RARE factor 8 and 6 averages). 35 axial slices with a slice thickness of 0.5 mm and no interslice distance were positioned to cover the whole brain. The field of view (FOV) was 3.5×3.5 cm and the matrix was 256×256 resulting in an inplane resolution of 137 µm. Calculation of lesion volume was carried out with the program Analyze 5.0 (Analyze Direct, Inc.; Lenexa USA) [13], [19] (Figure 3 C).

### Neurological score

Neurological evaluation was accomplished by a blinded observer 24 h and 48 h after MCAO using the 18-point neurological scoring system of Garcia et al [13], [20]. This neurological score will test for spontaneous activity, motor impairments and sensorial function. Severe impairments were graded 0 or 1, and no observed deficits were graded 3 (Figure 3D).

### Blood parameters

Blood samples were taken by retrobulbar puncture to determine oxygen- and carbon dioxide-partial pressure, pH, hematocrit, glucose-, sodium- and potassium concentration before and after MCAO (Table S1). Data were quantified using a RADIOMETER ABL 555 SERIES (Radiometer medical A/S, Copenhagen, Denmark).

### Nissl staining

**Figure 4:**
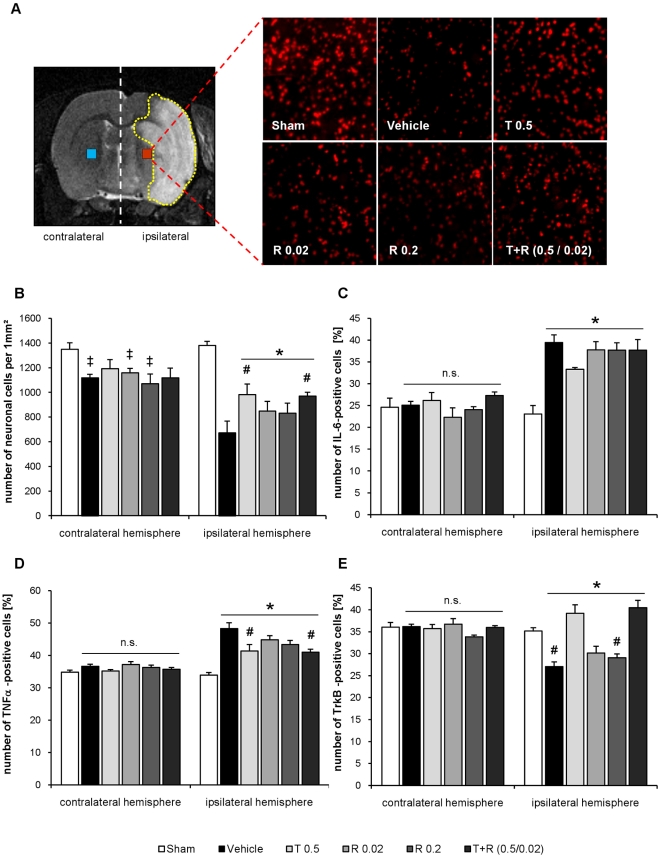
Intervention study. A) Typical MRI section showing infracted region (white area) from a Wistar rat 48 h after MCAO, the squares mentioned the counting area of the paraventricular penumbra in the ipsilateral and the correspondent area in the contralateral site. Representative pictures of Nissl staining of sham, vehicle, telmisartan 0.5 mg/kg (T 0.5), ramipril 0.02 mg/kg (R 0.02), ramipril 0.2 mg/kg (R 0.2) and combination of telmisartan 0.5 mg/kg and ramipril 0.02 mg/kg (T+R 0.5/0.02). B) Number of Nissl positive, viable neurons per mm^2^ in the periventricular penumbra and the correspondent contralateral site 48 h after MCAO. Columns from the left: sham, vehicle, telmisartan 0.5 mg/kg (T 0.5), ramipril 0.02 mg/kg (R 0.02), ramipril 0.2 mg/kg (R 0.2) and combination of telmisartan 0.5 mg/kg and ramipril 0.02 mg/kg (T+R 0.5/0.02). *: *p*<0.05 vs. sham; #: *p*<0.05 vs. vehicle C) Relative number of IL-6 positive cells (% of all cells) in the periventricular penumbra and the correspondent contralateral site 48 h after MCAO. Columns from the left: sham, vehicle, telmisartan 0.5 mg/kg (T 0.5), ramipril 0.02 mg/kg (R 0.02), ramipril 0.2 mg/kg (R 0.2) and combination of telmisartan 0.5 mg/kg and ramipril 0.02 mg/kg (T+R 0.5/0.02), counting area 0.4 mm^2^. *p<0.05 vs. sham D) Relative number of TNF-alpha positive cells (% of all cells) in the periventricular penumbra and the correspondent contralateral site 48 h after MCAO. Columns from the left: sham, vehicle, telmisartan 0.5 mg/kg (T 0.5), ramipril 0.02 mg/kg (R 0.02), ramipril 0.2 mg/kg (R 0.2) and combination of telmisartan 0.5 mg/kg and ramipril 0.02 mg/kg (T+R 0.5/0.02), counting area 0.4 mm^2^. *p<0.05 vs. sham; #*p*<0.05 vs. vehicle E) Relative number of TrkB positive cells (% of all cells) in the periventricular penumbra and the correspondent contralateral site 48 h after MCAO. Columns from the left: sham, vehicle, telmisartan 0.5 mg/kg (T 0.5), ramipril 0.02 mg/kg (R 0.02), ramipril 0.2 mg/kg (R 0.2) and combination of telmisartan 0.5 mg/kg and ramipril 0.02 mg/kg (T+R 0.5/0.02), counting area 0.4 mm^2^. *p<0.05 vs. sham; #*p*<0.05 vs. vehicle.

Rat brains were coronally sectioned into slices of 7 µm thickness in a cryostat and, subsequently, fixed in acetone for 5 min. For the staining of viable neurons sections were incubated with NeuroTrace (diluted 1∶50 in PBS) followed by DAPI staining (both from Molecular Probes, Eugene, Oregon, USA) according to the manufacturer's instructions. The area for counting the positively stained neurons was determined by comparing HE-stained sections and MRI sections of the animals of the experiment to choose an area of approx. 1 mm^2^ in the periventricular penumbra in all animals. In addition, the corresponding area in the contralateral hemisphere was investigated. Cells were counted by a blinded investigator (Figure 4A).

### Immunofluorescent staining

For staining of intracellular cytokines TNFα and IL-6, sections were blocked with 5% donkey serum (PAN Biotech GmbH, Aidenbach, Germany) and with 10% donkey serum for staining of TrkB, both diluted in PBS. Subsequently, the blocking buffer was removed and sections were incubated with primary antibodies (Santa Cruz Biotechnology Inc., Heidelberg, Germany; all diluted 1∶50 in blocking buffer) at 4°C, overnight. After incubation, primary antibodies were rinsed from sections three times with PBS. Cy3-conjugated secondary antibodies (1∶100 in 5% donkey serum in PBS, Chemicon, Schwalbach/Ts, Germany) were added and allowed to incubate for 1 hour at room temperature. The secondary antibodies were rinsed three times with PBS, and the sections were treated with 10 ng/ml DAPI for 15 min at room temperature. After washing for three times with PBS, coverslips were mounted on slides with mounting medium (Dako Cytomation, Hamburg, Germany). A Leica DM IRE2 microscope (Leica Microsystems, Wetzler, Germany) was used to generate the images. The counting area was determined by comparing HE-stained section and MRI sections of all animals of this experiment to choose an area (approximately 1 mm^2^ for the Nissl staining and 0.4 mm^2^ for the immunostaining) that was part of the penumbra in all animals. In this experiment, this area was in a periventricular location. In addition, the corresponding area in the contralateral hemisphere was investigated. Cells were counted by a blinded investigator (Figure 4B–E).

### Statistics

All data were analysed using SPSS 16.0 for Windows and presented as arithmetic mean ± standard error of the mean (SEM).

Analysis of variance by ANOVA-analysis followed post hoc tests (Scheffe'- or tukey-test). Differences between groups were compared by the Wilcoxon rank sum test for independent and the Wilcoxon signed rank sum test for dependant samples.

Survival was analyzed using Kaplan Meier analysis followed by log-rank test. Differences between two groups of interest were analyzed using the Mann-Whitney-U test. Results were considered significantly different at a value of p<0.05.

## Results

### Stroke prevention in SHR-SP

#### Systolic blood pressure in SHR-SP

Figure 1A shows systolic blood pressure (SBP) during treatment. SBP was comparable in all groups at the beginning of salt loading. The development of hypertension was prevented in rats treated with telmisartan, ramipril and their respective combination.

#### Course of treatment in SHR-SP

Figure 1A shows the course of treatment to achieve equal SBP between treatment groups (150 mmHg) during experiment. With the beginning of the high salt diet, SHR-SP were randomly assigned to four different treatment groups: group 1 served as control (untreated; *n* = 24), group 2 treated with the angiotensin AT1 receptor blocker telmisartan in the drinking water (initial dose T; 1.23 mg/kg bwt has to be raised up to 2.31 mg/kg bwt at week 6 and to 3.62 mg/kg bwt at the end of the study; *n* = 27), group 3 treated with the ACE-inhibitor ramipril in drinking water (initial dose R; 1.06 mg/kg bwt has to be raised up to 4.9 mg/kg bwt within week 6 and to 12.87 mg/kg bwt at the end of the study; *n* = 27), and group 4 was treated with a combination of telmisartan and ramipril in the drinking water (initial dose T; 0.25 mg/kg bwt plus R, 0.25 mg/kg bwt; has to be raised up to 0.57 mg/kg bwt each within week 6 and to 0.71 mg/kg bwt each at study end; n = 26). The dose of ramipril had to be increased considerably more to keep blood pressure at 150 mmHg than the dose of telmisartan. The respective doses of T and R in combination therapy were relatively low and ineffective as monotherapy.

#### Neurological score of SHR-SP

Figure 1B shows the development of the neurological deficits over time and indicates the number of animals remaining in each group at the corresponding time point. Neurological scores were comparable in all groups at the beginning of the protocol. The development of neurological deficits was decelerated in rats treated with telmisartan, ramipril or their combination. At 4 weeks, vehicle-treated animals began to develop neurological signs associated with stroke: piloerection, jumping, aggression, prostration, loss of body symmetry and convulsions. Rats treated with telmisartan, ramipril or their combination did not display such neurological signs until the end of the study.

#### Survival rate in SHR-SP

Survival rates of animals are summarized in Figure 1C. Fifty percent of the animals in the vehicle-treated group died within 13 weeks of salt loading. Telmisartan, ramipril and combination treatment equally improved survival: within the 13 week duration of the study, all animals in the treated groups survived.

#### Stroke verification in SHR-SP

The brains of dead SHR-SP rats and, after finishing the experiment, the brains of survived animals were analysed by MRI. All dead SHR-SP animals had suffered from stroke, and the survived animals showed no signs of oedema, ischemic or haemorrhagic lesions. See representative pictures Figure 1D.

### Acute intervention study in normotensive rats

#### Mean arterial blood pressure during MCAO

Blood pressure was monitored before MCAO in conscious rats, and during and after MCAO/reperfusion under anaesthesia. Mean arterial blood pressure remained unchanged and not different from vehicle-treated controls under the established pre-treatment doses of telmisartan and ramipril (low dose) or their combination. In contrast, pre-treatment with ramipril at 0.2 mg/kg bwt (high dose) significantly reduced mean arterial blood pressure before, during and after MCAO (Figure 3A).

#### Blood parameters

Oxygen, carbon dioxide, pH, glucose, hematocrit, sodium, potassium showed no significant differences between groups during MCAO (Table S1).

#### Cerebral blood flow

During MCAO, all animals showed a significant reduction in CBF in comparison to baseline conditions. After withdrawal of the occluding filament, ipsilateral blood flow was restored to approximately 90% of baseline levels. Reductions in CBF during MCAO and during reperfusion were identical in vehicle- and drug-treated groups (Figure S3B).

#### Body weight

After stroke, body weight decreased significantly in the vehicle-treated group in comparison to sham operated animals. In the telmisartan and combination pre-treated groups, but not in the ramipril-pre-treated group, body weight was not reduced and remained in the range of the sham operated animals (Figure 3B).

#### Infarct volume

Cerebral ischemic areas were visualized by the MRI technique and normalized to body weight. On day 2 after MCAO, the size of ischemic lesions was 150 mm^3^/100 g bwt. on average in vehicle pre-treated animals with MCAO. Telmisartan- and combination pre-treatment, but not ramipril pre-treatment, significantly reduced the mean volume of the ischemic area (Figure 3C).

#### Neurological deficits

In all stroke groups, a significant reduction in the Garcia score was recorded 24 h and 48 h after MCAO compared to sham operated animals (Figure 3D). Neurological outcome was significantly improved in the telmisartan- and combination-treated group after 24 h as well as 48 h after MCAO. In the ramipril-treated groups the improvement did not reach statistical significance (Figure 3D).

#### Mortality

In accordance with the literature [21]–[23], mortality rate within the first 24 h after MCAO was between 28% and 30% in all stroke groups.

#### Neuronal cell loss

For histological assessment of neuronal damage in the periventricular penumbra, Nissl staining was performed 48 h after MCAO. In injured neurons, the Nissl substance breaks apart. Compared with sham operation the number of Nissl positive cells was markedly reduced in the ipsilateral penumbra after MCAO (*p*<0.05), as shown in Figure 4A. Neuronal loss of the ischemic periventricular penumbra was significantly improved in telmisartan- and combination- but not in ramipril pre-treated groups (*p*<0.05). The corresponding contralateral brain region showed a significant reduction of neuronal cells in stroke-vehicle and ramipril treated groups in comparison to sham operated animals (*p*<0.05) (Figure 4B).

#### IL-6 and TNF-α by Immunohistochemistry

The proinflammatory cytokines, IL-6 and TNF-α, were significantly increased 48 h after MCAO in comparison to sham operated animals.

In both telmisartan- and combination pre-treated groups, protein levels of TNF-α in the ipsilateral penumbra were significantly decreased in comparison to the stroke- vehicle group, whereas IL-6 remained unchanged in all pre-treatment groups. The expression of TNF-α and IL-6 in the corresponding contralateral brain region was not different from the one in sham operated animals (Figure 4C–D)

#### TrkB by Immunohistochemistry

The BDNF receptor, TrkB, was significantly down-regulated in the ipsilateral penumbra 48 h after MCAO in the vehicle-treated group in comparison to sham operated animals. Pre-treatment with telmisartan and the combination increased, on protein level, the expression of BDNF receptor TrkB in comparison to stroke-vehicle group. The expression of receptor TrkB in the corresponding contralateral brain region was not different from the one in sham operated animals (Figure 4E).

## Discussion

Stroke is one of the leading causes of death and invalidity in the modern world [24]. Consequently, prevention and treatment of stroke have become major issues in experimental and clinical research. Compared to other antihypertensive drugs, angiotensin AT1-receptor blockers (ARBs) have been demonstrated to be effective in primary and secondary stroke prevention in several clinical studies [2]–[6], [25] even when blood-pressure reduction was similar between different study arms.

In contrast, in The Profess trial on secondary stroke, AT1-receptor blockade did not confer additional benefit when compared to other stroke therapies. However, interpretation of these findings is limited by to the short observation period [26].

The ONTARGET trial compared the efficacy of the angiotensin receptor antagonist, telmisartan, the angiotensin-converting enzyme inhibitor, ramipril, and their combination in reducing cardio- and cerebrovascular risk [6]. The study population included patients with or without hypertension, with coronary artery disease, stroke, peripheral vascular disease or diabetes with end-organ damage but excluded patients with congestive heart failure. Telmisartan was not inferior to ramipril with respect to the primary endpoint including stroke incidence, and the combination of the two drugs was not superior to each one alone [6].

Concerning stroke prevention, the present results in salt-fed stroke-prone SHR are in line with those of ONTARGET. With doses adjusted to achieve equal antihypertensive actions, telmisartan, ramipril or their combination all prevented stroke incidence and stroke-related neurological deficits during the 13 weeks of observation, while untreated hypertensive controls had a mortality of 50% at the end and experienced stroke-related neurological deficits during the course of the study. Like in the ONTARGET trial, it thus appears that in hypertensive individuals, the blood pressure lowering drug actions determine stroke incidence with no difference between principles of RAS inhibition.

Interestingly, during the course of treatment, the dose of ramipril had to be increased considerably more than the corresponding dose of telmisartan to keep blood pressure at 150 mmHg, and the dose of each compound in combination therapy was much lower than the respective monotherapy doses.

However, stroke incidence, as much as it is part of the combined endpoint in many cardiovascular drug trials, constitutes a relatively crude measure of drug-induced brain protection since it is mainly related to the vascular, but not to the neuronal part of the stroke issue. The latter also includes potential neuroprotective drug effects limiting stroke extension and reducing post-ischemic neuronal damage.

In this regard, a number of animal studies have suggested that the beneficial effects of ARBs in stroke include a blood pressure–independent element of neuroprotection, and some of the underlying molecular mechanisms have already been described [14]–[18], [26]–[29]. With respect to the ACE inhibitors, the experimental and clinical literature presents rather equivocal data on blood pressure related- or unrelated effects in stroke [1], [11], [20], [30].

In the second part of the present study, we therefore compared potential blood pressure-independent neuroprotective effects of systemic pre-treatment with telmisartan, ramipril and their combination after focal brain ischemia induced by MCAO with reperfusion in normotensive rats. General criteria of comparative dose definition were equi-potency with respect to persistent blockade of the peripheral RAS in vivo (inhibition of the pressor responses to intravenous Ang I and AngII, respectively) and absence of blood pressure lowering effects before, during and after focal brain ischemia.

Given these experimental conditions, pre-treatment with telmisartan alone and with the combination of telmisartan and ramipril proved to be more effective than ramipril in reducing stroke volume, loss of body weight and neurological deficits. Combination treatment with telmisartan plus ramipril seems to be not superior to telmisartan alone.

These findings are in line with those of a previous experimental head-to-head comparison involving candesartan and ramipril [20] and with most of the animal studies on potential neuroprotective effects of RAS inhibitors [14]–[18], [20], [27]–[29]. Since cerebral blood flow was reduced during MCAO to the same extent with no differences between vehicle-treatment and drugs, mechanisms other then those merely related to brain perfusion have to be responsible for differences in neuroprotection between ARBs and ACE inhibitors.

One simple explanation for our findings could be that ARBs in general, and telmisartan or candesartan in particular [9], [31], have a higher propensity to cross the blood-brain barrier (BBB) than ACE inhibitors, eg. ramipril, [10] and to interfere with the brain RAS, i.e. inhibit the actions of non-vascular AT1 receptors and stimulate the actions of unopposed AT2 receptors inside the BBB [9]. Indeed, it has been demonstrated that AT2 receptors inside the brain contributes to the neuroprotective effects (reduced infarct volume and neurological deficits, improved neuronal survival) of central AT1 receptor blockade after MCAO [18], which is also underlined by the severe outcome after MCAO in AT2 receptor KO mice [29] and smaller lesion area and much larger penumbra in AT1 receptor KO mice [28]. Under this assumption, combined treatment with telmisartan and ramipril would reflect the central effects of telmisartan but afford no further benefits by ACE inhibition beyond AT1 receptor blockade.

Further mechanisms of neuroprotection that may be related to central AT1 receptor blockade include the neurotrophin receptor TrkB which showed an enhanced expression under telmisartan and combination therapy in the penumbra of ischemic brain tissue. Analyzing BDNF- and TrkB knockout mice, this neurotrophin system has been identified to be essential for the development and survival of several distinct neuronal populations *in vitro* and also *in vivo* [32]–[34]. We observed an exclusive increase of the TrkB protein in the telmisartan- and combination group compared to the other groups with brain ischemia. This finding, reflecting an intensified interaction of BDNF with its specific receptor, TrkB, points to a local, molecular mechanism of neuroprotective actions which may have contributed to the reduced neuronal loss in the ischemic penumbra under telmisartan- and combination treatment as observed in the present study.

### Conclusion

In salt-fed SHR-SP equi-antihypertensive treatment with telmisartan, ramipril or their combination completely prevented neurological deficits and stroke occurrence pointing to blood pressure reduction as the leading mechanism in stroke prevention. Conversely, in acute stroke intervention in normotensive rats, sub-antihypertensive pre-treatment with telmisartan and combination treatment was significantly more effective than ramipril alone in terms of neuroprotection. The combination of telmisartan and ramipril seems to be not superior to telmisartan alone in this experimental setting.

## Supporting Information

Figure S1 **Study design.** A) Prevention study Baseline measurements were performed at the age of 9 weeks as described in Material and Methods. Subsequently, all rats were switched to a high salt diet and randomly assigned to four different treatment groups. The initial drug doses are indicated. Individual drug doses were selected on the basis of achieving equal blood pressure (150 mmHg) throughout the protocol. Several parameters were estimated in various time-points as indicated in the figure. B) Intervention study In a pilot study, the appropriate treatment doses of the drugs were determine as described in Material and Methods. Based on the dose finding, animals were randomly allocated to five different treatment groups. Several parameters were estimated in various time-points as indicated in the figure.
(TIF)

Figure S2 **Pilot study.** A) Effects of different doses of telmisartan or vehicle administered subcutaneously on the pressor responses to intravenously injected angiotensin II (50 ng/kg bw n = 7 per group). B) Effects of different doses of ramipril or vehicle administered subcutaneously on the pressor responses to intravenously injected angiotensin I (150 ng/kg bw; n = 7).
(TIF)

Figure S3 **Intervention study.** A) Magnetic resonance angiography of the rat brain from a ventral view before MCAO, during occlusion and after reperfusion on cerebral perfusion. On the left: middle cerebral artery before cerebral ischemia was induced; in the middle: during occlusion is the MCA in the circle not visible; on the right: the apparent MCA after reperfusion. B) Changes in the rCBF in the zone of ischemia before, during and after occlusion of the middle cerebral artery for 90 minutes and during the reperfusion period in rats treated subcutaneously with vehicle (white bars; n = 12), or telmisartan (light grey bars; n = 13) or ramipril (grey bars; n = 11) or combination of telmisartan and ramipril (black bars; n = 8) on 5 consecutive days before the induction of ischemia. rCBF values (mean ±SD) are expressed as the percentage of baseline values recorded before occlusion of the middle cerebral artery. In all groups the reduction of rCBF during occlusion was significant reduced *p<0.05.
(TIF)

Table S1 Arterial blood oxygen, carbon dioxide, pH values, glucose, haematocrite, sodium and potassium concentration before middle cerebral artery occlusion in rats treated subcutaneously with vehicle (V), telmisartan (T = 0.5 mg/kg), or ramipril (R1 = 0.01 mg/kg bw; R2 = 0.1 mg/kg bw) or combination telmisartan and ramipril (T = 0.5 mg/kg bw and R1 = 0.01 mg/kg bw).
(TIF)
