# Supplementary figures and images for: Prevention and Intervention Studies with Telmisartan, Ramipril and Their Combination in Different Rat Stroke Models
Source: PLoS One. 2011 Aug 25;6(8):e23646. doi: 10.1371/journal.pone.0023646 (PMC3161992; doi:10.1371/journal.pone.0023646)

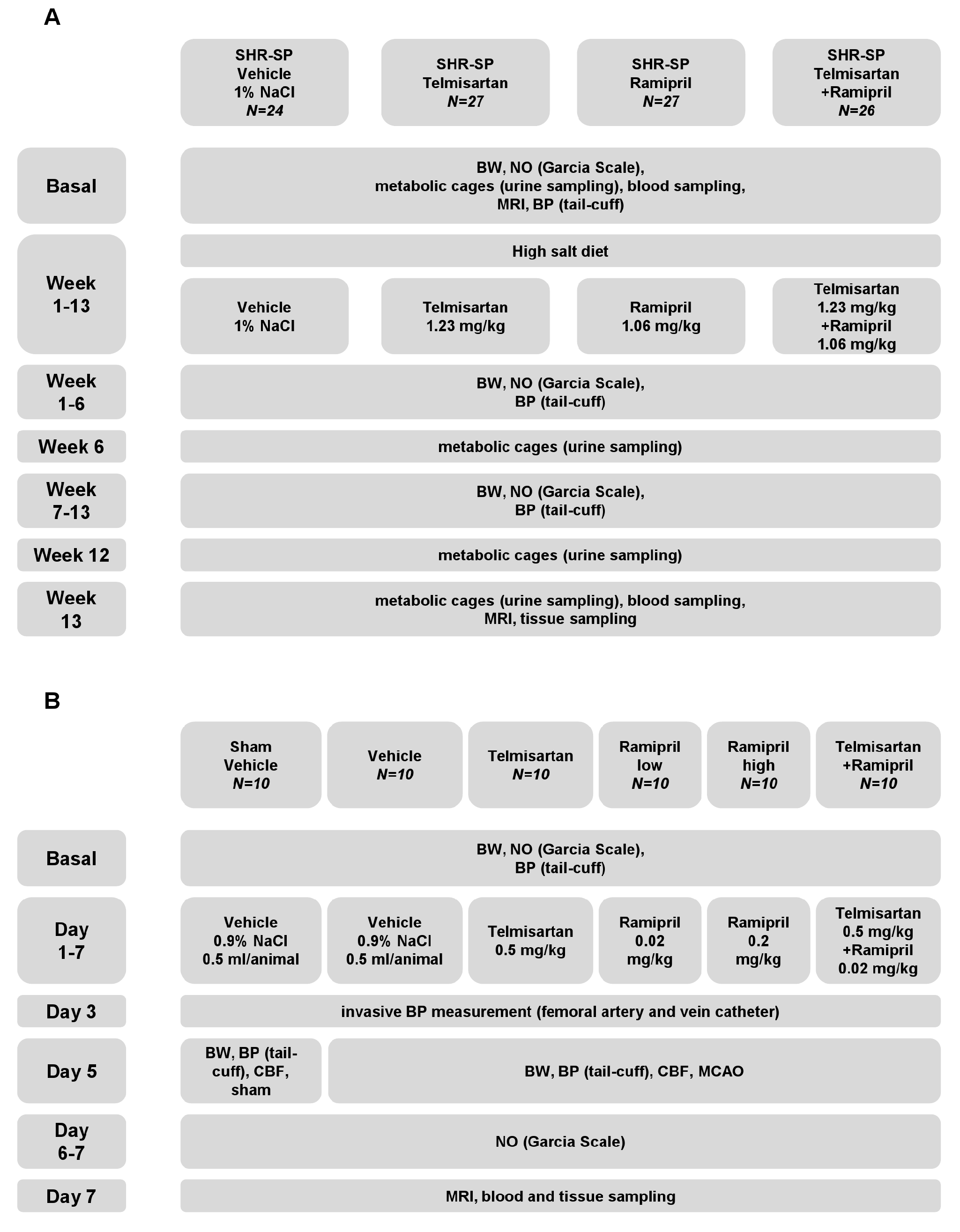

Supplement: Figure S1 — Study design. A) Prevention study Baseline measurements were performed at the age of 9 weeks as described in Material and Methods. Subsequently, all rats were switched to a high salt diet and randomly assigned to four different treatment groups. The initial drug doses are indicated. Individual drug doses were selected on the basis of achieving equal blood pressure (150 mmHg) throughout the protocol. Several parameters were estimated in various time-points as indicated in the figure. B) Intervention study In a pilot study, the appropriate treatment doses of the drugs were determine as described in Material and Methods. Based on the dose finding, animals were randomly allocated to five different treatment groups. Several parameters were estimated in various time-points as indicated in the figure. (TIF) [file pone.0023646.s001.tif]

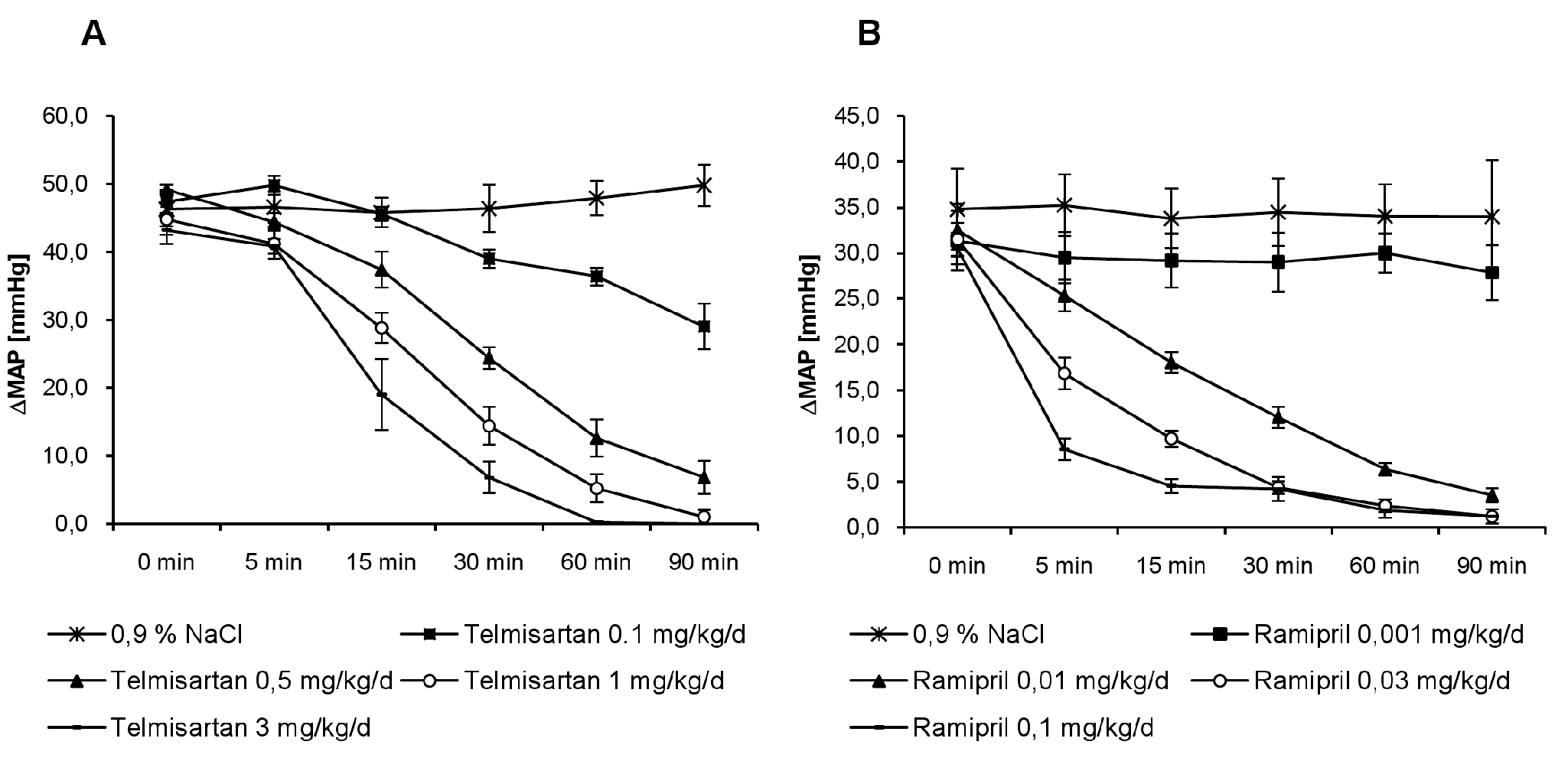

Supplement: Figure S2 — Pilot study. A) Effects of different doses of telmisartan or vehicle administered subcutaneously on the pressor responses to intravenously injected angiotensin II (50 ng/kg bw n = 7 per group). B) Effects of different doses of ramipril or vehicle administered subcutaneously on the pressor responses to intravenously injected angiotensin I (150 ng/kg bw; n = 7). (TIF) [file pone.0023646.s002.tif]

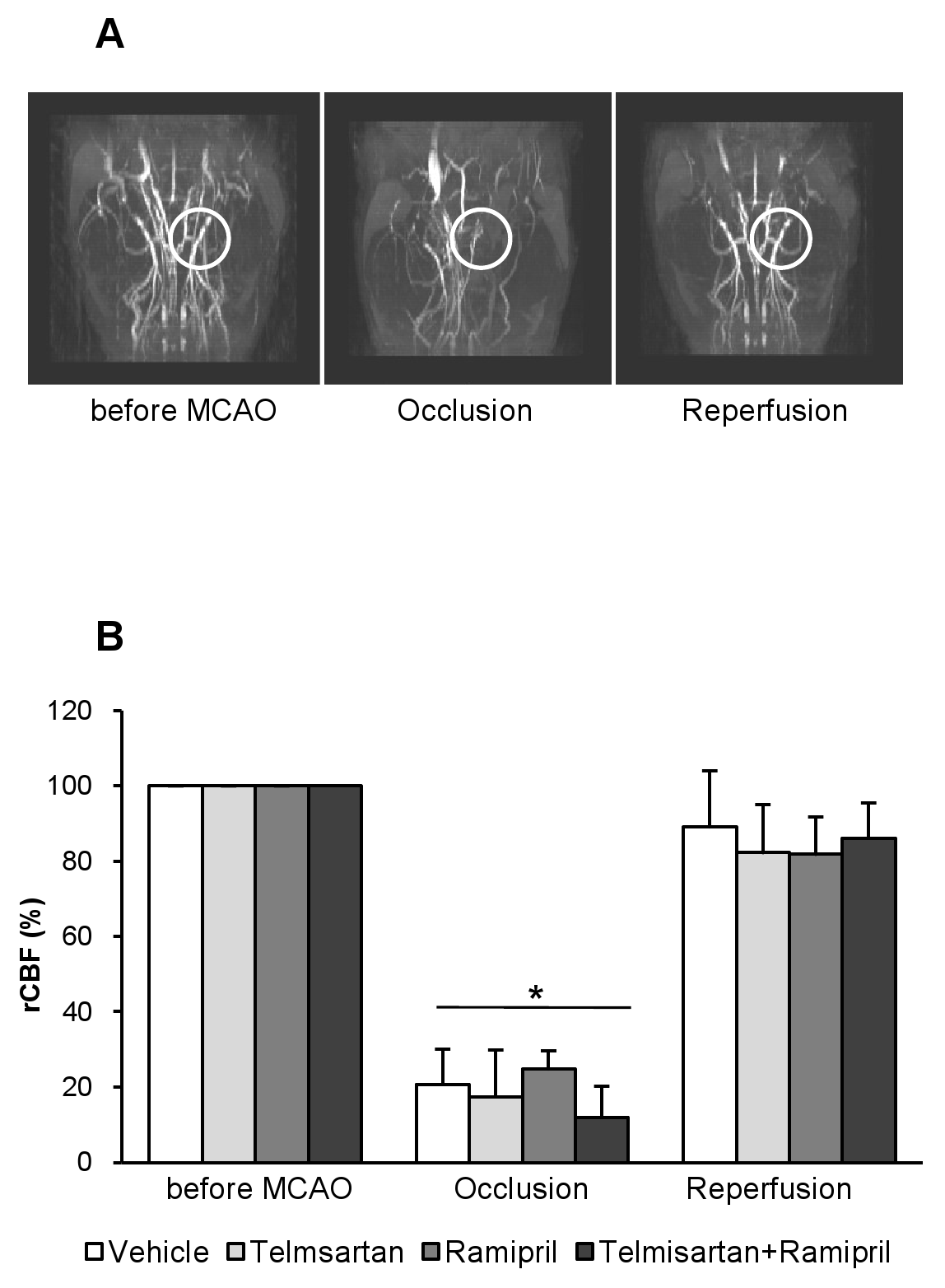

Supplement: Figure S3 — Intervention study. A) Magnetic resonance angiography of the rat brain from a ventral view before MCAO, during occlusion and after reperfusion on cerebral perfusion. On the left: middle cerebral artery before cerebral ischemia was induced; in the middle: during occlusion is the MCA in the circle not visible; on the right: the apparent MCA after reperfusion. B) Changes in the rCBF in the zone of ischemia before, during and after occlusion of the middle cerebral artery for 90 minutes and during the reperfusion period in rats treated subcutaneously with vehicle (white bars; n = 12), or telmisartan (light grey bars; n = 13) or ramipril (grey bars; n = 11) or combination of telmisartan and ramipril (black bars; n = 8) on 5 consecutive days before the induction of ischemia. rCBF values (mean ±SD) are expressed as the percentage of baseline values recorded before occlusion of the middle cerebral artery. In all groups the reduction of rCBF during occlusion was significant reduced *p<0.05. (TIF) [file pone.0023646.s003.tif]

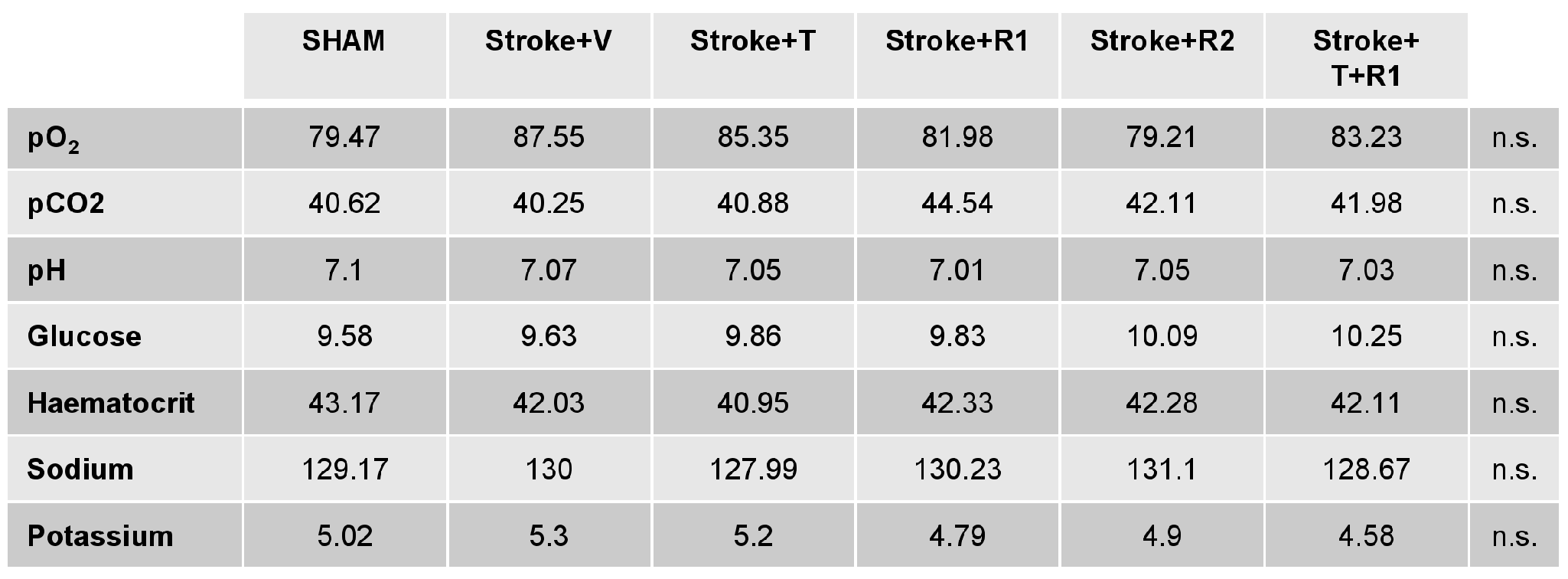

Supplement: Table S1 — Arterial blood oxygen, carbon dioxide, pH values, glucose, haematocrite, sodium and potassium concentration before middle cerebral artery occlusion in rats treated subcutaneously with vehicle (V), telmisartan (T = 0.5 mg/kg), or ramipril (R1 = 0.01 mg/kg bw; R2 = 0.1 mg/kg bw) or combination telmisartan and ramipril (T = 0.5 mg/kg bw and R1 = 0.01 mg/kg bw). (TIF) [file pone.0023646.s004.tif]
